# Betamethasone use and risk factors for pulmonary edema during the perinatal period: a single-center retrospective cohort study in Japan

**DOI:** 10.1186/s12884-022-04918-2

**Published:** 2022-08-12

**Authors:** Takashi Nishikawa, Ken Fukuhara

**Affiliations:** Department of Obstetrics and Gynecology, Kurashiki Central Hospital, Ohara Health Care Foundation, 1-1-1 Miwa, Kurashiki, Okayama 7108602 Japan

**Keywords:** Pulmonary edema, Antenatal corticosteroid, Betamethasone, Tocolytic agent, Ritodrine hydrochloride, Magnesium sulfate

## Abstract

**Background:**

A few studies have reported that maternal administration of antenatal corticosteroids increased the risk of pulmonary edema (PE). However, despite the increasing usage rate of betamethasone as antenatal corticosteroid, maternal administration of betamethasone as a risk factor for PE has not been well studied. This study aimed to evaluate how maternal backgrounds and complications, tocolytic agents, and betamethasone affect the incidence of PE during the perinatal period and determine the risk factor for PE.

**Methods:**

This was a single-center retrospective cohort study in Kurashiki, Japan. The study subjects were patients who had been admitted to our hospital for perinatal management including pregnancy, delivery and puerperium between 2017 and 2020. The primary outcome measure was defined as the incidence of PE during hospitalization. First, in all study subjects, Cox proportional hazards model was used to determine the risk factor for PE during the perinatal period. Next, using propensity score matching, we divided the patients into the betamethasone and betamethasone-free groups and examined the association between betamethasone use and the incidence of PE with Cox proportional hazards model.

**Results:**

During the study period, 4919 cases were hospitalized, and there were 16 PE cases (0.3%). In all analyzed subjects, the occurrence of PE was significantly associated with preeclampsia (hazard ratio 16.8, 95% confidence intervals (CI) 5.39–52.7, *P* < 0.001) and the combined use of the tocolytic agents such as ritodrine hydrochloride and magnesium sulfate, and betamethasone (hazard ratio 11.3, 95% CI 2.66–48.1, *P* = 0.001). In contrast, after propensity score matching, no statistically significant difference was found between the betamethasone and betamethasone-free groups in the incidence of PE (hazard ratio 3.19, 95% CI 0.67–15.3, *P* = 0.145).

**Conclusions:**

A combined use of tocolytic agents and antenatal corticosteroids such as betamethasone may be an independent risk factor for PE during the perinatal period. On the other hand, betamethasone use alone may not be associated with the incidence of PE. When tocolytic agents and betamethasone are administrated to pregnant women, it is important to pay attention to the appearance of maternal respiratory symptoms.

## Background

Acute pulmonary edema (PE) during pregnancy and the postpartum period is uncommon and its frequency is estimated to be approximately 0.08–0.5% [1, 2, 3]. When it occurs, however, termination of pregnancy and admission to the intensive care units (ICU) needs to be considered. According to Williams Obstetrics [1], the etiology of PE during the perinatal period is classified as cardiogenic and noncardiogenic; the former is hydrostatic edema due to increased pulmonary capillary pressure, while the latter is osmotic edema due to damage to the capillary endothelium and alveolar epithelium. Pregnancy with heart disease is known to be one of the risk factors for PE, but PE during pregnancy is usually noncardiogenic [1, 2]. Regarding noncardiogenic PE, in addition to the conditions that promote activation of vascular endothelial cells such as preeclampsia and sepsis, the use of tocolytic agents is known to be a risk factor for PE. There are many reports documenting an association between tocolytic agents and PE, including those from Japan, where continuous intravenous infusion of tocolytic agents is often performed over 1 week [3, 4, 5].

Also, maternal administration of antenatal corticosteroids has been reported to increase the risk of maternal PE [6, 7]. In particular, the risk may be increased by the concomitant use of tocolytic agents such as ritodrine hydrochloride and magnesium sulfate, multiple pregnancies, and hypertensive disorder in pregnancy [8, 9].

It is well known that antenatal corticosteroid therapy reduces the incidence of neonatal respiratory distress syndrome and intraventricular hemorrhage by accelerating fetal lung maturation [10]. In Japan, the guidelines recommend intramuscular administration of betamethasone 12 mg every 24 hours for a total of two doses if preterm delivery is expected within 1 week after 24 weeks of gestation and before 34 weeks of gestation [11, 12], and the usage rate of betamethasone has been increasing [13]. However, most reports only mentioned a possible association between antenatal corticosteroids and PE [2–, 7, 8, 9], and despite the increasing usage rate of betamethasone, maternal administration of betamethasone as a risk factor for PE has not been well studied.

Because our hospital is a tertiary perinatal center where many high-risk pregnant women are hospitalized for preterm labor, we often use betamethasone as an antenatal corticosteroid, and encountered PE cases after administration of betamethasone, which can lead to termination of pregnancy and admission to the ICU. We therefore hypothesized that betamethasone used would be a risk factor for PE during the perinatal period and designed the clinical study. The aim of this study is to determine the risk factors for PE during the perinatal period.

## Methods

### Study design and population

This was a single-center retrospective cohort study using medical records and conducted at Kurashiki Central Hospital, which is a tertiary perinatal center in Kurashiki, Japan. The study subjects were patients who had been admitted to our hospital for perinatal management including pregnancy, delivery and puerperium between January 2017 and December 2020. We regarded different pregnancies in the same woman as different cases. In addition, even if the patient was hospitalized multiple times during the same pregnancy, the treatment during each hospitalization such as pregnancy and delivery management was counted separately, and analysis was performed on a case basis.

### Data collection

We reviewed subjects’ medical records and collected the following items: maternal age, weeks of gestation, body mass index, parity, and number of fetuses on admission, occurrence of delivery, duration of hospitalization, and obstetric complication (heart disease including arrhythmia and valvular disease, hypertensive disorder in pregnancy including preeclampsia, preterm rupture of membrane, and gestational diabetes mellitus). Moreover, the use of tocolytic agents (ritodrine hydrochloride and magnesium sulfate) and that of betamethasone as an antenatal corticosteroid, the incidence of PE, and period from admission or administration of these agents to occurrence of PE or discharge, and admission to the ICU were assessed.

### Clinical assessment and management

PE was diagnosed with clinical symptom of dyspnea, decreased percutaneous oxygen saturation, enhanced pulmonary vascular shadows or decreased permeability of the entire lung field on chest plain radiographs. As mentioned above, PE during the perinatal period is classified as cardiogenic and noncardiogenic, and the etiologies are endothelial activation with capillary-alveolar leakage and myocardial failure with hydrostatic edema from excessive pulmonary capillary pressure, respectively. The former includes cardiomyopathy and left-sided valvular disease, while the latter includes preeclampsia, sepsis, and use of tocolytic agents. The diagnoses of hypertensive disorder in pregnancy, preeclampsia, and gestational diabetes mellitus was made according to the guidelines [11, 12, 14, 15]. Tocolytic agents were used to delay when frequent uterine contractions and shortening of the uterine cervix to less than 25 mm in length or dilation of the uterine cervix to 20 mm or more were observed on admission due to imminent preterm birth, premature rupture of membrane, and chorioamnionitis. In Japan, ritodrine hydrochloride and magnesium sulfate are approved and widely used as tocolytic agents within insurance coverage [11, 12]. Ritodrine hydrochloride was used after 16 weeks of gestation and magnesium sulfate after 22 weeks of gestation. Magnesium sulfate was used when ritodrine hydrochloride could not be used because of side effects or when ritodrine hydrochloride could not suppress uterine contractions. Magnesium sulfate was also used to prevent eclampsia. The dosage of each drug was adjusted by obstetricians, paying attention to the improvement of symptoms and the appearance of adverse effects, but the administration was sometimes continued for 1 week or longer. Intramuscular administration of betamethasone 12 mg every 24 hours for a total two doses was performed if preterm delivery was expected within 1 week after 24 weeks of gestation and before 34 weeks of gestation.

### Primary and secondary outcome measure

The primary outcome measure was defined as the incidence of PE during hospitalization. The secondary outcome measure was the time from the day of hospital admission to the onset of PE. Cases in which PE did not occur were censored on the day of hospital discharge.

### Statistical analysis

All statistical analyses were performed with EZR (Jichi Medical University Saitama Medical Center, Saitama, Japan) which is a graphical user interface for R (R Foundation for Statistical Computing, Vienna, Austria). More precisely, it is a modified version of R commander (version 1.6–3) that is designed to add statistical functions frequently used in biostatistics [16]. All *P*-values were two sided and *P*-values of 0.05 or less were considered statistically significant.

First, the analysis of the baseline patient characteristics was performed. Continuous variables were expressed as median and interquartile ranges, and categorical variables as the numbers of patients and percentages. With the presence or absence of PE as the dependent variable, continuous variables were examined using Mann-Whitney’s U-test, and categorical variables using Fisher’s exact tests or Chi-square test. Because ritodrine hydrochloride, magnesium sulfate, and betamethasone are often used in combination with each drug, and there were also no PE cases caused by betamethasone use alone, we classified the patients into three groups: drug-free group, tocolytic agents group (ritodrine hydrochloride, magnesium sulfate, or both), and tocolytic agents + betamethasone group.

Second, in all study subjects, risk factors for PE were examined by multivariate survival analysis. Using the time from the day of hospital admission to the onset of PE as the time variable, risk factors for PE were extracted by the Cox proportional hazards model, and hazard ratios, 95% confidence intervals (CI), and *P*-values were calculated. Moreover, we used propensity score matching to minimize the effect of co-variables that affect betamethasone use and adjust confounding factors. Multiple logistic regression analysis was used to calculate propensity scores using the following variables: age, body mass index, and parity on admission, multiple pregnancies, heart disease, preeclampsia, preterm rupture of membrane, gestational diabetes mellitus, and use of ritodrine hydrochloride and magnesium sulfate. The nearest neighbor method with a caliper value of 0.2 was used, and matching ratio is 1:1. After matching, the Kaplan-Meier method was used to generate survival curves for the betamethasone and betamethasone-free groups, and the log-rank method to test for significant differences. Then, hazard ratios, 95% CI, and *P*-values for betamethasone use were calculated using the Cox proportional hazards model with the time form the day of hospital admission to the onset of PE as the time variable.

## Results

### Baseline characteristics and clinical outcomes

During the study period, 4919 cases were hospitalized. Table 1 shows the baseline patient characteristics and clinical outcomes by the presence or absence of PE. There were 16 PE cases (0.3%). As complications, hypertensive disorder in pregnancy including preeclampsia, multiple pregnancies were significantly more common in the PE group than in the non-PE group. Of 16 PE cases, there were 9 cases with hypertensive disorder in pregnancy, 8 of which had preeclampsia. Four cases were multiple pregnancies, all of which were twin pregnancies. The usage rates of betamethasone, ritodrine hydrochloride, and magnesium sulfate, and the rate of admission to the ICU were significantly higher in the PE group than in the non-PE group. In the PE group, ritodrine hydrochloride was administrated to 5, magnesium sulfate to 12, and betamethasone to 10. There were no significant differences in age and body mass index, parity on admission, preterm rupture of membrane, and gestational diabetes mellitus.

**Table 1:** Characteristics and clinical outcomes of the study population between non-pulmonary and pulmonary edema groups

	Non-PE group	PE group	*P*-values
	*n* = 4903	*n* = 16	
Background			
Maternal age, median [IQR]	32.0 [28.0, 36.0]	32.5 [30.0, 37.3]	0.28
Body mass index, median [IQR]	24.0 [22.0, 27.0]	24.0 [23.0, 26.0]	0.986
Nulliparity, n (%)	2292 (46.9)	11 (68.8)	0.13
Multiple pregnancies, n (%)	255 (5.2)	4 (25.0)	0.008
Number of fetuses			
1, n (%)	4631 (94.5)	12 (75.0)	0.018
2, n (%)	261 (5.3)	4 (25.0)	
3, n (%)	10 (0.2)	0 (0.0)	
4, n (%)	1 (0.0)	0 (0.0)	
Complications			
History of heart disease, n (%)	46 (0.9)	0 (0.0)	1
Hypertensive disorder in pregnancy, n (%)	356 (7.3)	9 (56.2)	< 0.001
Preeclampsia, n (%)	154 (3.1)	8 (50.0)	< 0.001
Gestational diabetes mellitus, n (%)	217 (4.4)	0 (0.0)	1
Preterm labor with rupture of membrane, n (%)	186 (3.8)	1 (6.2)	0.463
Drug			
Ritodrine hydrochloride, n (%)	557 (11.4)	5 (31.2)	0.029
Magnesium sulfate, n (%)	398 (8.1)	12 (75.0)	< 0.001
Betamethasone, n (%)	273 (5.6)	10 (62.5)	< 0.001
Combination use			
Free, n (%)	4022 (82.0)	3 (18.8)	NA
Ritodrine hydrochloride, n (%)	50 (1.0)	0 (0.0)	
Magnesium sulfate, n (%)	211 (4.3)	3 (18.8)	
Betamethasone, n (%)	63 (1.3)	5 (31.2)	
Ritodrine hydrochloride + Magnesium sulfate, n (%)	349 (7.1)	0 (0.0)	
Ritodrine hydrochloride+Betamethasone, n (%)	84 (1.7)	1 (6.2)	
Magnesium sulfate+Betamethasone, n (%)	48 (1.0)	0 (0.0)	
all drugs, n (%)	76 (1.6)	4 (25.0)	
Duration of ritodrine hydrochloride, median [IQR]	1.0 [1.0, 15. 5]	13.0 [3.0, 26.0]	0.204
Duration of magnesium sulfate, median [IQR]	2.0 [2.0, 4.0]	5.0 [3.0, 9.25]	0.008
Admission to ICU, n (%)	30 (0.6)	10 (62.5)	< 0.001

Values are presented as median [interquartile range] and numbers (percentage)

*PE* Pulmonary edema, *IQR* Interquartile range, *ICU* Intensive care unit

Table 2 shows the characteristics of the16 PE cases. Eight cases complicated preeclampsia. Although none of the 16 cases had a history of heart disease, 2 cases developed cardiogenic PE; one was caused by perinatal cardiomyopathy and the other by mitral regurgitation due to mitral valve prolapse during pregnancy. Of 16 cases, 14 cases developed PE during pregnancy and 2 case after delivery. The median week of gestation at the onset of PE was 31 + 2 weeks. Eleven cases underwent termination of pregnancy within 1 day of the onset of PE, while 3 cases were treated conservatively. In the 16 PE cases, there were no PE cases due to betamethasone use alone, but there were 10 PE cases due to the use of tocolytic agents + betamethasone. The median time from the start of ritodrine hydrochloride and magnesium sulfate to the onset of PE was 4 days and 2 days, respectively, and that from the last dose of betamethasone to the onset of PE was 0 days.

**Table 2:** Characteristics and clinical outcomes of pulmonary edema group

	PE group
	*n* = 16
Cause of PE	
Cardiogenic, n (%)	2 (12.5)
Noncardiogenic, n (%)	14 (87.5)
Onset of PE (gestational age), median [IQR]	31 + 2 [25 + 5, 34 + 1]
Pregnancy, n (%)	14 (87.5)
Postpartum, n (%)	2 (12.5)
Duration from admission to onset of PE (days), median [IQR]	3.0 [1.8, 7.3]
Duration from onset of PE to delivery (days), median [IQR]	0 [0.0, 1.0]
0 days, n (%)	9 (64.3)
1 day, n (%)	2 (14.3)
8 days, n (%)	1 (7.1)
10 days, n (%)	1 (7.1)
24 days, n (%)	1 (7.1)
Combination of use	
Free, n (%)	3 (18.8)
Tocolytic agents, n (%)	3 (18.8)
Tocolytic agents+betamethasone, n (%)	10 (62.5)
Duration from administration of ritodrine hydrochloride to onset of PE (days), median [IQR]	4.0 [4.0, 31.0]
Duration from administration of magnesium sulfate to onset of PE (days), median [IQR]	2.0 [1.0, 5.0]
Duration from last administration of betamethasone to onset of PE (days), median [IQR]	0 [0.0, 1.0]

Values are presented as median [interquartile range] and numbers (percentage)

*PE* Pulmonary edema, *IQR* Interquartile range, *ICU* Intensive care unit

### Risk factors for PE in all analyzed subjects

In all analyzed subjects, risk factors for PE were evaluated by the Cox proportional hazards model with PE as the dependent variable. The proportional hazards assumption for the model holds with *P-*values> 0.05. Table 3 shows the results of the Cox proportional hazards model. The occurrence of PE was significantly associated with preeclampsia (hazard ratio 16.8, 95% CI 5.39–52.7, *P* < 0.001) and the combined use of ritodrine hydrochloride, magnesium sulfate, and betamethasone (hazard ratio 11.3, 95% CI 2.66–480, *P* = 0.001).

**Table 3:** Results of multivariate survival analysis with Cox proportional hazard model

	Hazard ratio	95%CI	*P*-values
Multiple pregnancies	2.34	0.73–7.50	0.151
Preeclampsia	16.8	5.39–52.7	<0.001
Tocolytic agents	1.51	0.26–8.58	0.646
Tocolytic agents + betamethasone	11.3	2.66–48.1	0.001

Hazard ratio was estimated with Cox proportional hazard model adjusting for exposure time for relevant variables, and adjusted for preeclampsia, multiple pregnancies, and a combination use of tocolytic agents and betamethasone

*CI* Confidence intervals

### Risk factors for PE in all analyzed subjects by propensity score matching

**Fig. 1:**
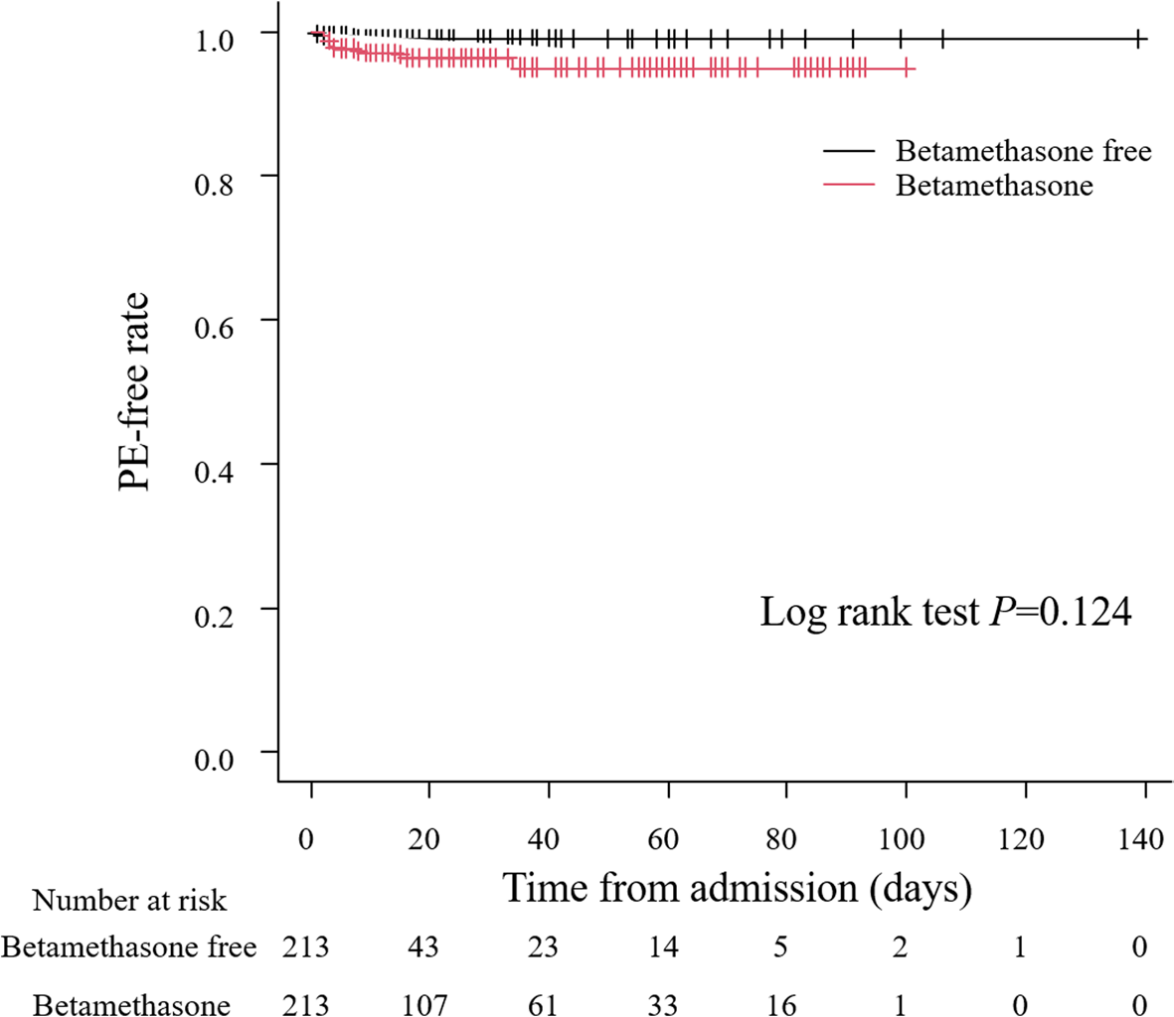
Kaplan-Meier curves for onset of pulmonary edema between betamethasone group and betamethasone-free group after propensity score matching

Tables 4 and 5 show a comparison of the baseline patient characteristics between the betamethasone and betamethasone-free groups before and after propensity score matching. Before propensity score matching, since betamethasone was used when the risk of preterm labor arouse, there were significant differences in the usage rates of ritodrine hydrochloride and magnesium sulfate, and also in the incidence rates of preeclampsia and preterm rupture of membrane between the two groups, as well as in body mass index, while there were no significant differences in maternal age and parity on admission. After propensity score matching, the standardized difference between the betamethasone and betamethasone-free groups was less than 0.1 excluding nulliparity and multiple pregnancies, suggesting a balanced population. Figure 1 shows the Kaplan-Meier curves for PE in the two groups after propensity score matching. There was no significant difference in the survival curves between the two groups (*P* = 0.124). On the basis of the results of the Cox proportional hazards model, betamethasone was not regarded as a significant risk factor PE (hazard ratio 3.19, 95%CI 0.67–15.3, *P* = 0.145) (Table 6).

**Table 4:** Characteristics and clinical outcomes before propensity score matching between betamethasone group and betamethasone-free group

	Betamethasone-free group	Betamethasone group	*P*-values	SD
	*n* = 4636	*n* = 283		
Background				
Maternal age, median [IQR]	32.0 [28.0, 36.0]	33.0 [29.0, 36.0]	0.14	0.083
Body mass index, median [IQR]	25.0 [22.0, 27.0]	23.0 [21.0, 25.0]	< 0.001	0.022
Nulliparity, n (%)	2176 (47.0)	127 (45.0)	0.539	0.04
Multiple pregnancies, n (%)	218 (4.7)	41 (14.5)	< 0.001	0.337
Number of fetuses				
1, n (%)	4401 (94.9)	242 (85.5)	< 0.001	0.343
2, n (%)	231 (5.0)	34 (12.0)		
3, n (%)	3 (0.1)	7 (2.5)		
4, n (%)	1 (0.0)	0 (0.0)		
Complications				
Heart disease, n (%)	46 (1.0)	0 (0.0)	0.111	0.142
Hypertensive disorder in pregnancy, n (%)	322 (6.9)	43 (15.2)	< 0.001	0.265
Preeclampsia, n (%)	133 (2.9)	29 (10.2)	< 0.001	0.301
Gestational diabetes mellitus, n (%)	202 (4.4)	15 (5.3)	0.454	0.044
Preterm labor with rupture of membrane, n (%)	120 (2.6)	67 (23.7)	< 0.001	0.657
Drug				
Ritodrine hydrochloride, n (%)	397 (8.6)	165 (58.3)	< 0.001	1.241
Magnesium sulfate, n (%)	262 (5.7)	148 (52.3)	< 0.001	1.199
Duration of Magnesium sulfate (days), median [IQR]	2.0 [2.0, 3.0]	3.0 [2.0, 9.0]	< 0.001	0.463
Duration of Ritodrine hydrochloride (days), median [IQR]	1.0 [1.0, 7.0]	15.0 [4.0, 37.0]	< 0.001	0.704
Clinical outcomes				
PE, n (%)	6 (0.1)	10 (3.5)	< 0.001	0.256
Duration from last administration of betamethasone to onset of PE(days), median [IQR]	2.0 [2.0, 2.0]	2.5 [1.8, 3.0]	0.68	NA
Admission to ICU, n (%)	29 ( 0.6)	11 (3.9)	< 0.001	0.221

Values are presented as median [interquartile range] and numbers (percentage)

*PE* Pulmonary edema, *IQR* Interquartile range, *ICU* Intensive care unit, *SD* Standardized difference

**Table 5:** Characteristics and clinical outcomes after propensity score matching between betamethasone group and betamethasone-free group

	Betamethasone-free group	Betamethasone group		
	*n* = 211	*n* = 211	*P*-values	SD
Background				
Maternal age, median [IQR]	32.0 [28.0, 36.0]	32.0 [29.0, 36.0]	0.745	0.025
Body mass index, median [IQR]	23.0 [21.5, 26.0]	23.0 [21.0, 26.0]	0.798	0.002
Nulliparity, n (%)	92 (43.6)	96 (45.5)	0.769	0.038
Multiple pregnancies, n (%)	28 (13.3)	27 (12.8)	1	0.041
Number of fetuses				
1, n (%)	183 (86.7)	184 (87.2)	0.925	0.052
2, n (%)	26 (12.3)	24 (11.4)		
3, n (%)	2 (0.9)	3 (1.4)		
4, n (%)	0 (0.0)	0 (0.0)		
Complications				
History of heart disease, n (%)	0 (0.0)	0 (0.0)	1	< 0.001
Hypertensive disorder in pregnancy, n (%)	51 (24.2)	38 (18.0)	0.152	0.151
Preeclampsia, n (%)	36 (17.1)	26 (12.3)	0.216	0.134
Gestational diabetes mellitus, n (%)	10 (4.7) (15.3)	12 (5.7)	0.827	0.043
Preterm labor with rupture of membrane, n (%)	35 (16.6)	38 (18.0)	0.797	0.038
Drug				
Ritodrine hydrochloride, n (%)	101 (47.9)	105 (49.8)	0.77	0.038
Magnesium sulfate, n (%)	100 (47.4)	93 (44.1)	0.558	0.067
Clinical outcomes				
PE, n (%)	1 (0.5)	8 (3.8)	0.037	0.231

Values are presented as median [interquartile range] and numbers (percentage)

*PE* Pulmonary edema, *IQR* Interquartile range, *ICU* Intensive care unit, *SD* Standardized difference

**Table 6:** Results of multivariate survival analysis with Cox proportional hazard model after propensity score matching

	Hazard ratio	95%CI	*P*-values
Use of betamethasone	3.19	0.67–15.3	0.145

Hazard ratio was estimated with Cox proportional hazard model

*CI* Confidence intervals

## Discussion

The results of this study suggested that betamethasone use in combination with tocolytic agents was an independent risk factor for PE. In contrast, the results of the adjustment for the confounders by propensity score matching suggested that betamethasone use alone was not a significant risk factor for PE.

First, this study showed that betamethasone used in combination with tocolytic agents was an independent risk factor for PE during the perinatal period, as well as preeclampsia. In Japan, ritodrine hydrochloride and magnesium sulfate are generally used as tocolytic agents. Ritodrine hydrochloride causes maternal tachycardia and retention of sodium and water due to activation of the renin-angiotensin-aldosterone system [2, 5]. Also, magnesium sulfate increases hydrostatic pressure and decreases permeability of pulmonary capillaries [17]. Prolonged administration of these drugs has been often performed, especially in Japan, which increases the risk of PE [4]. On the other hand, in the literature, there was no study of how betamethasone use alone can increase the risk of PE, except for that of Ogunyemi. Ogunyemi reported that the use of tocolytic agents and antenatal corticosteroid between 24 and 33 weeks of gestations were significantly associated with the risk of PE with an odds ratio 4.3 (95% CI 2.3–8.4, *P* < 0.001) and 2.3 (95% CI 1.3–4.0, *P* = 0.002), respectivly [6]. The study showed tocolytic agents and betamethasone was a significant risk factor for PE during pregnancy, whereas the current study was performed for the patients who admitted to our hospital for the purpose of perinatal management including pregnancy, delivery, and puerperium, and showed that betamethasone used in combination with tocolytic agents was an independent risk factor for PE.

Second, the results of the adjustment for the confounders by propensity score matching suggested that betamethasone use alone was not a significant risk factor for PE. As mentioned above, Ogunyemi suggested betamethasone use was a significant risk factor for PE during pregnancy, and the results were inconsistent. In this study, however, there were also no PE cases caused by betamethasone use alone, and it is difficult to judge the significance from the result of this study. Although corticosteroids such as betamethasone at high doses have the effect of water retention, they uncommonly have an adverse effect on healthy people [18]. Regarding maternal effects of antenatal corticosteroids, the Cochran review does not show increased risks of chorioamnionitis, endometritis, and maternal death, and states that they can be used relatively safely [10]. This study did not reveal that the use of antenatal corticosteroids could increase the risk of PE, which is consistent with previous reports, but further study is necessary for the risk assessment of betamethasone use alone.

Another finding of this study was that the preeclampsia and multiple pregnancies were significantly more common in PE cases than in non-PE cases, as well as the usage of tocolytic agents and betamethasone. Also, multivariate analysis revealed that preeclampsia was a risk factor for PE. Preeclampsia is known to the cause of noncardiogenic PE [1, 3], which is consistent with the result of previous studies. On the other hand, multiple pregnancies themselves were not the independent risk of PE. Maternal cardio output in multiple pregnancies increased by about 20% compared to single pregnancy, suggesting that multiple pregnancies are associated with the risk of PE [2, 19], whereas, there is a report documenting that multiple pregnancies in combination with the use of tocolytic agents increase the risk of PE [20]. Thus, the result of this study revealed that multiple pregnancies were not a risk factor for PE. Furthermore, there were no significant differences in body mass index and pregnancy with heart disease between PE and non-PE cases. Regarding obesity, although it has been pointed out that obesity is a risk factor for PE [1, 2], there is a study showing that no significant difference was observed in the incidence of PE between obese pregnant women complicated with preeclampsia and non-obese pregnant women [21], which supports the results of this study. Regarding pregnancy with heart disease, this may be related to the fact that there were no pregnant women with high-risk heart disease in this study, such as a history of NYHA 3 or higher heart failure, which increases the risk of PE, and that the incidence of PE in pregnant women with heart disease has been reported to be on the decline [22].

To best of our knowledge, this is first report from Japan that the combined use of antenatal corticosteroids and tocolytic agents may be an independent risk factor for PE during the perinatal period. Prolonged use of tocolytic agents such as ritodrine hydrochloride and magnesium sulfate and the incidence of preeclampsia, which are risk factors for PE, has been recently increasing in Japan with an increase in pregnancy in advanced maternal age [23]. According to the database of the neonatal research network of Japan, the usage rate of antenatal corticosteroid has increased from 2003 to 2012 [13]. The results of this study therefore suggest that the combination use of tocolytic agents and betamethasone in perinatal management requires caution in Japan, because PE during the perinatal period may lead to termination of pregnancy and admission to the ICU due to the change of general condition. In this study, 10 of 16 PE cases were admitted to the ICU, which was significantly more than those in non-PE cases. As a prediction of that, this study, which revealed that the use of tocolytic agents and betamethasone is a risk factor for PE, is considered to be important for perinatal management.

The major limitation of this study is its small sample size. PE during the perinatal period is relatively uncommon, and increasing the number of research cooperative facilities and securing an appropriate sample size were challenging. Another limitation is that we did not investigate the treatment and prognosis of PE cases. Further studies to examine them are warranted.

## Conclusions

A combined use of tocolytic agents such as ritodrine hydrochloride and magnesium sulfate, and antenatal corticosteroids such as betamethasone may be an independent risk factor for PE during the perinatal period. Also, betamethasone use alone may not be associated with the incidence of PE. When a combined use of tocolytic agents and betamethasone is administrated to pregnant women, it is important to pay attention to the appearance of maternal respiratory symptoms.

## Data Availability

The datasets generated and/or analysed during the current study are not publicly available due to the huge amount of data but are available from the corresponding author on reasonable request.

## Abbreviations

PE: Pulmonary edema
ICU: Intensive care unit
CI: Confidence intervals
